# Tocilizumab-induced mucosal injury in the terminal ileum mimicking intestinal Behçet’s disease: A case report

**DOI:** 10.1097/MD.0000000000034118

**Published:** 2023-06-23

**Authors:** Sotaro Ozaka, Masahide Fukuda, Haruhiko Takahashi, Koshiro Tsutsumi, Masao Iwao, Yuka Hirashita, Kensuke Fukuda, Kazuhisa Okamoto, Mie Arakawa, Ryo Ogawa, Mizuki Endo, Kazuhiro Mizukami, Naganori Kamiyama, Takashi Kobayashi, Masaaki Kodama, Kazunari Murakami

**Affiliations:** a Department of Gastroenterology, Faculty of Medicine, Oita University, Oita, Japan; b Department of Infectious Disease Control, Faculty of Medicine, Oita University, Oita, Japan.

**Keywords:** adult-onset Still disease, case report, enteritis, intestinal ulcer, tocilizumab

## Abstract

Tocilizumab, a humanized anti-interleukin-6 (IL-6) receptor monoclonal antibody, is used for the treatment of adult-onset Still disease (AOSD). Despite its efficacy in many clinical situations, concerns have been raised regarding intestinal mucosal injury in patients receiving tocilizumab.

**Patient concerns:**

A 64-year-old woman with a history of AOSD was admitted to our hospital with hematochezia. She had AOSD for 15 years and underwent treatment with biweekly tocilizumab 9 months prior to admission. Colonoscopy revealed a large punched-out ulcer in the terminal ileum. On pathological evaluation, nonspecific enteritis with lymphocytes and eosinophils were seen. Based on the location and shape of the lesion, we suspected intestinal Behçet’s disease. However, the ulcer reduced in size over time by discontinuation of tocilizumab without additional drug treatment, indicating that it was a drug-induced ulcer.

**Diagnosis:**

The patient was diagnosed with tocilizumab-induced small intestinal ulcer.

**Interventions:**

The patient treated with the discontinuation of tocilizumab.

**Outcomes:**

The discontinuation of tocilizumab resulted in ulcer scarring. There was no recurrence of hematochezia.

**Lessons:**

Tocilizumab can cause deep ulcerative lesions in the terminal ileum, which may resemble intestinal Behçet’s disease. It is important to continuously monitor abdominal symptoms during tocilizumab therapy and aggressively perform colonoscopy when hematochezia or abdominal pain is observed.

## 1. Introduction

Tocilizumab is a humanized anti-interleukin-6 (IL-6) receptor monoclonal antibody that exerts strong anti-inflammatory effects by blocking IL-6 signaling. It is used to treat not only autoinflammatory diseases, including rheumatoid arthritis (RA) and adult-onset Still disease (AOSD), but also severe coronavirus disease 2019 (COVID-19). While tocilizumab is a highly effective treatment for these diseases,^[1,2]^ it has been reported to cause gastrointestinal mucosal injury, such as lower gastrointestinal perforation.^[3]^ In particular, tocilizumab increases the risk of intestinal perforation in patients with prior diverticulitis.^[4]^ Schiff et al reported that out of 4009 RA patients treated with tocilizumab, 26 developed gastrointestinal perforation, with perforation occurring in the colon of 18 patients. Of these 18 patients with colon perforation, 16 (88.8%) had acute perforations that complicated colonic diverticular disease.^[5]^ In contrast, several cases of tocilizumab-induced mucosal injury unrelated to diverticulitis have also been reported.^[6–9]^ Although previous reports have indicated that tocilizumab increases the risk of gastrointestinal perforation, the pathogenesis of tocilizumab-induced mucosal injury remains largely unknown. In addition, most cases of tocilizumab-induced mucosal injury are diagnosed as a result of intestinal perforation due to diverticulitis; therefore, few cases have allowed for detailed endoscopic observation of the lesions. Herein, we report a case of tocilizumab-induced small intestinal ulcer. Interestingly, the tocilizumab-induced lesion observed in the present case was a deep punched-out ulcer resembling intestinal Behçet’s disease in the terminal ileum. The lesion was discovered as a result of hematochezia and successfully treated with the discontinuation of tocilizumab.

## 2. Case presentation

**Figure 1.**
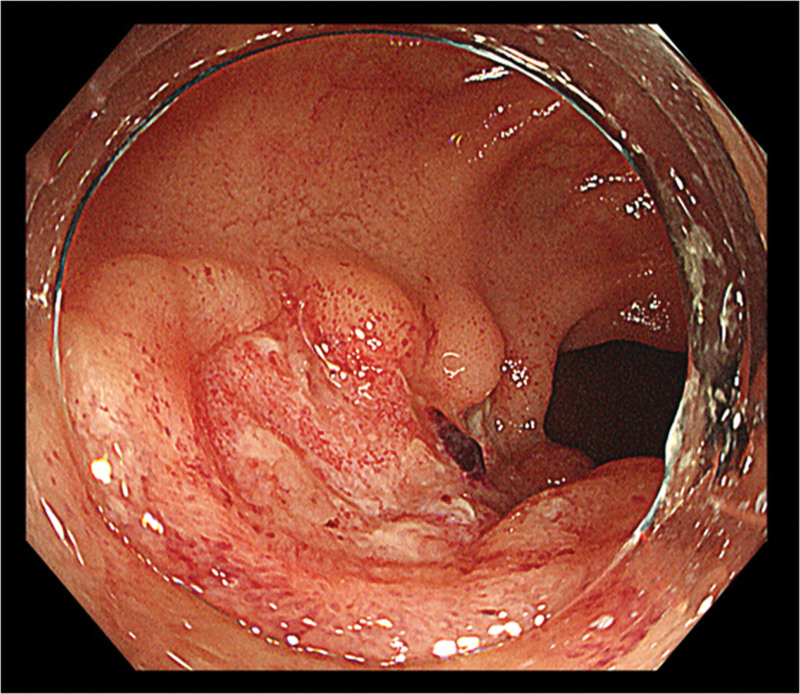
Colonoscopy reveals a round punched-out ulcer in the terminal ileum.

**Figure 2.**
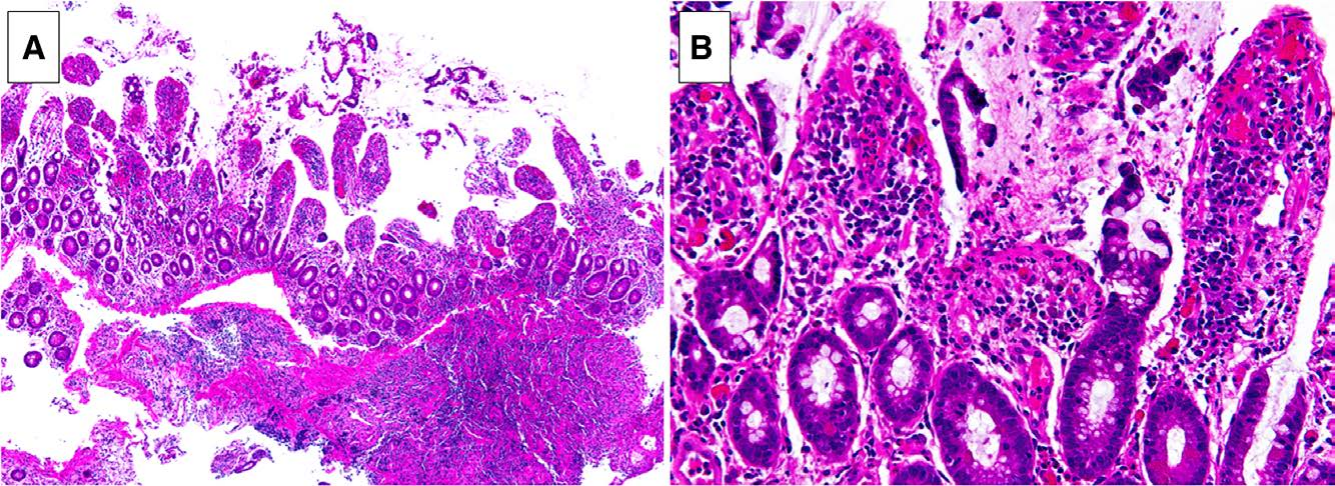
(A) Low power field. (B) High power field. Hematoxylin-eosin staining of biopsy specimen taken from terminal ileum were shown. Erosive findings were observed in the epithelium. nonspecific enteritis with lymphocytes and eosinophils were seen.

A 64-year-old woman with a history of AOSD, mitral regurgitation, and paroxysmal atrial fibrillation was admitted to a local hospital with hematochezia. She was on regular prednisolone, cyclosporine, vonoprazan, levothyroxine, rivaroxaban, sulfamethoxazole trimethoprim, and alfacalcidol. Colonoscopy revealed fresh blood covering the terminal ileum wall. Two days later, she was admitted to our hospital with suspected small intestinal bleeding. Her blood pressure was 127/76 mm Hg, heart rate was 60 bpm with sinus rhythm, and body temperature was 36.7°C. An abdominal examination revealed mild tenderness of the lower abdomen. Laboratory tests revealed the following results: White blood cell count, 6990/μL; hemoglobin, 11.7 g/dL; blood urea nitrogen 17.6 mg/dL; creatinine level of 0.83 mg/dL; C-reactive protein, 0.03 mg/dL (Table 1). Abdominal contrast-enhanced computed tomography showed no mass or vascular lesions, and there was no extravasation of the contrast agent in the delayed phase. Esophagogastroduodenoscopy revealed atrophic gastritis but no bleeding lesions. Colonoscopy revealed a large, deep, round punched-out ulcer in the terminal ileum (Fig. 1). An exposed vessel was observed at the base of the ulcer. On pathological evaluation, erosive findings were observed in the epithelium, and nonspecific enteritis with lymphocytes and eosinophils were seen (Fig. 2). There were no findings of ischemic colitis, cytomegalovirus, granuloma, small intestine cancer, or malignant lymphoma. Bacterial cultures of the fecal samples and ileal mucosa showed no specific findings. An antigenemia test for cytomegalovirus and an interferon-gamma release assay for tuberculosis were both negative. Based on the location and shape of the lesion, we suspected intestinal Behçet’s disease.

**Table 1:** Laboratory data.

<Blood cell count>	Data	Reference	<Biochemical examination>	Data	Reference
White blood cells	6990/μL	3300–8600	Proteinase-3-anti-neutrophil cytoplasmic antibody	(−)	(−)
Red blood cells	377 × 10^4^/μL	386-492 × 10^4^	Myeloperoxidase-anti-neutrophil cytoplasmic antibody	(−)	(−)
Blood hemoglobin	11.7 g/dL	11.6–14.8	Antinuclear antibody	<40	<40
Platelet count	23.2 × 10^4^/μL	15.8–34.8	Antimitochondrial antibody	(−)	(−)
<Biochemical examination>			<Infectious screening>		
Total protein	6.4 g/dL	6.6–8.1	Hepatitis B virus antigen	(−)	(−)
Albumin	4.2 g/dL	4.1–5.1	Hepatitis C virus antibody	(−)	(−)
Blood urea nitrogen	17.6 mg/dL	8–20	Cytomegarovirus antigen (C7 HRP)	(−)	(−)
Creatinine	0.83 mg/dL	0.46–0.79	T-SPOT	(−)	(−)
Total-bilirubin	1.8 mg/dL	0.4–1.5			
Glucose	129 mg/dL	73–109	Human Leukocyte Antigen-B51	(−)	(−)
Aspartate aminotransferase	57 U/L	13–30	Human Leukocyte Antigen-A26	(−)	(−)
Alanine aminotransferase	47 U/L	7–23			
Lactate dehydrogenase	340 U/L	124–222	<Tumor marker>		
γ-glutamyl transpeptidase	18 U/L	9–32	Carbohydrate antigen 19–9	9.0 U/mL	<37
Sodium	139 mEq/L	138–145	Carcinoembryonic antigen	<1.7	<5
Chloride	103 mEq/L	101–108			
Potassium	4.1 mEq/L	3.6–4.8			
C-reactive protein	0.03 mg/dL	0–0.14			

**Figure 3.**
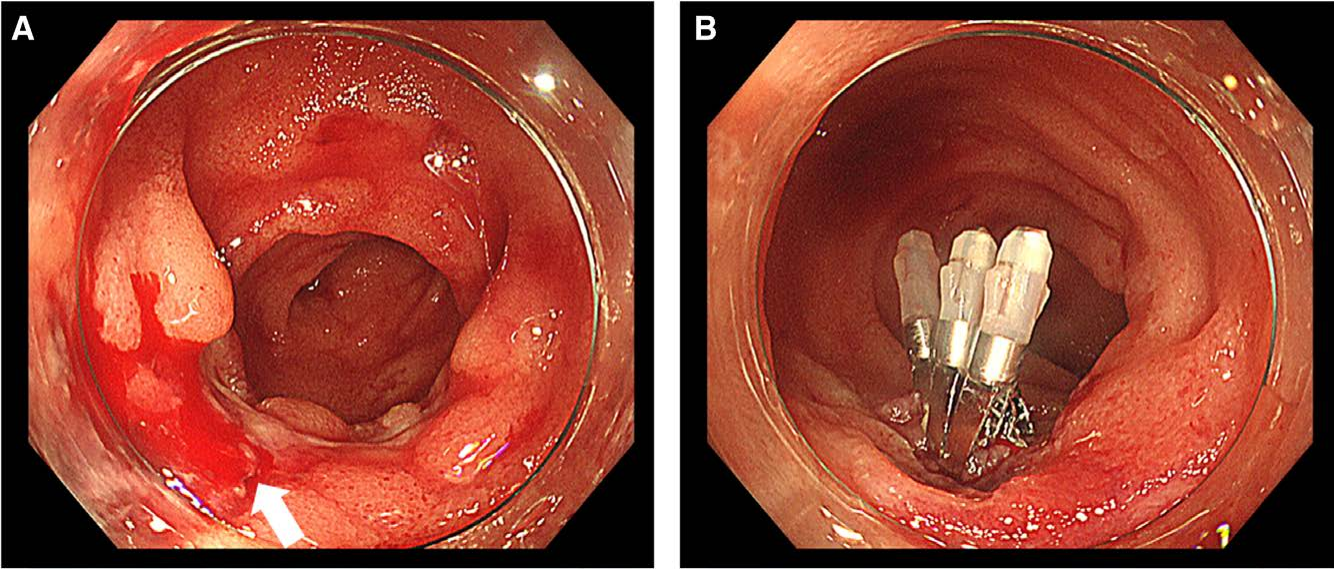
(A) Urgent colonoscopy shows active bleeding from an exposed vessel at the base of the ulcer in the terminal ileum (arrow). (B) Hemostatic treatment is performed using clip placement.

**Figure 4.**
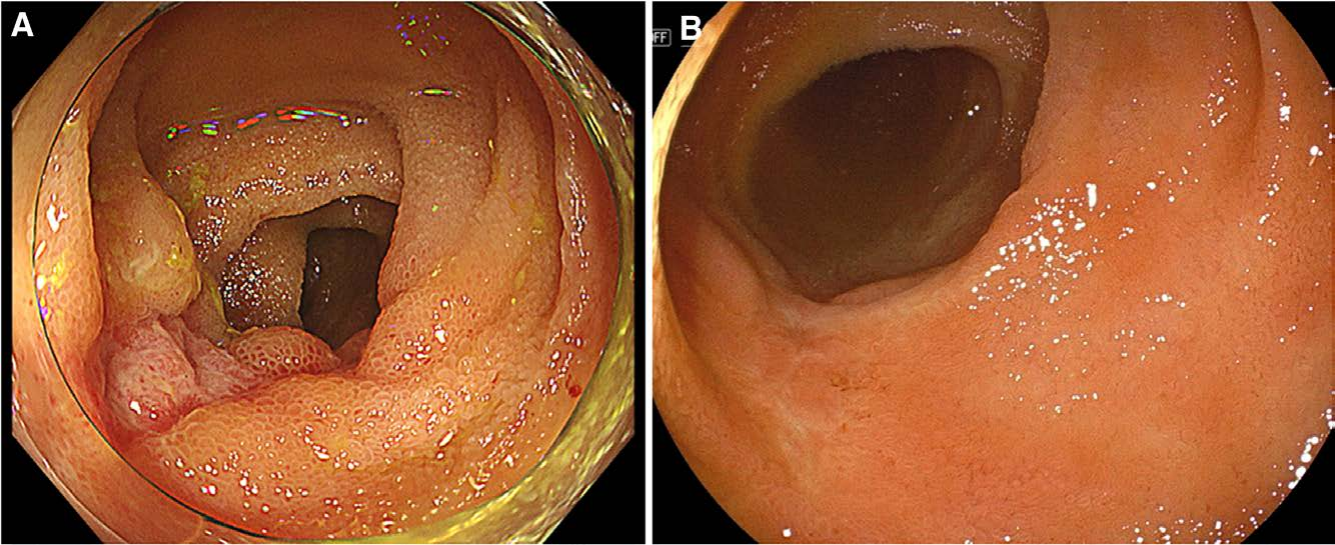
(A) After the discontinuation of tocilizumab, the ulcer is smaller than before. (B) Colonoscopy performed 2 mo after discharge reveals scarred ulcer.

The patient presented with massive hematochezia 3 days after admission. An emergency colonoscopy revealed no active bleeding from the ileal ulcer. Subsequent capsule endoscopy to detect other lesions and bleeding points showed no further small bowel involvement other than the ulcer in the terminal ileum. Rivaroxaban was discontinued and fasting was initiated. However, the patient presented with massive hematochezia 3 days later. Urgent colonoscopy revealed active bleeding from the protruding vessel at the base of the ulcer in the terminal ileum, which was successfully treated with clip placement (Fig. 3). Additionally, we noticed that the ulcer size had reduced compared with that the initial examination without additional drug treatment. In addition to regular medications mentioned previously, treatment with biweekly tocilizumab was initiated 9 months prior to admission. The last dose of tocilizumab was administered 7 days prior to the current presentation. As tocilizumab-induced mucosal injury was suspected, tocilizumab was discontinued. A follow-up colonoscopy performed 7 days after hemostatic treatment showed that the ulcer had shrunk further (Fig. 4A). The patient was discharged from the hospital without further recurrence of bleeding. Colonoscopy performed 2 months after discharge revealed that the ulcer had healed (Fig. 4B). The discontinuation of tocilizumab resulted in ulcer scarring, which finally led to the diagnosis of a tocilizumab-induced small intestinal ulcer. There was no recurrence of hematochezia. The patient is still undergoing maintenance treatment for AOSD with prednisolone and cyclosporine. Written informed consent for the publication of this case report, including any accompanying images, was obtained from both the patient and her next of kin.

## 3. Discussion

Our experience highlighted 2 important clinical points. First, tocilizumab can cause deep ulcerative lesions in the terminal ileum resembling intestinal Behçet’s disease. Second, in cases of properly diagnosed tocilizumab-induced ulcers, unnecessary immunosuppressive therapy should be avoided and tocilizumab should be discontinued.

Tocilizumab, a humanized anti-IL-6 receptor monoclonal antibody, is used to treat various autoimmune diseases such as RA and AOSD.^[1,2]^ AOSD is an autoinflammatory disorder of unknown etiology characterized by high spiking fever, rash, arthritis, and lymphadenopathy.^[10]^ As recent studies have shown the importance of IL-6 in its pathogenesis, tocilizumab is now used for the treatment of refractory AOSD.^[11]^ Our patient had AOSD for 15 years and underwent treatment with various medications, including prednisolone and cyclosporine; however, these therapies could not control her disease. Therefore, tocilizumab was initiated.

Despite its efficacy in many clinical conditions, concerns have been raised regarding intestinal mucosal injury in patients receiving tocilizumab therapy.^[3]^ Gastrointestinal perforation has recently been reported as a rare but potentially life-threatening side effect of tocilizumab. In one study, the analysis of clinical trials evaluating the safety of tocilizumab in RA patients reported that of 4009 patients undergoing treatment with tocilizumab, 26 developed gastrointestinal perforation, with perforation occurring in the colon in 18 patients. Of these 18 patients with colon perforation, 16 had acute perforations that complicated colonic diverticular disease.^[5]^ The current estimated incidence of tocilizumab-induced gastrointestinal perforation is approximately 2.7 to 3.0/1000 patient-years.^[4,10]^ A retrospective cohort study by Xie et al showed that the overall incidence rate of gastrointestinal perforation was significantly higher in RA patients receiving tocilizumab than in patients treated with other medications, including tofacitinib, abatacept, rituximab, and anti-tumor necrosis factors.^[12]^ In particular, the risk of lower intestinal perforation is significantly elevated with tocilizumab therapy, and an important risk factor for lower intestinal perforation associated with tocilizumab is previous diverticulitis.^[4]^ Although the mechanism of action of tocilizumab in the etiology of diverticular perforation remains unclear, it has been hypothesized that elevated C-reactive protein and abdominal pain caused by diverticulitis are masked by tocilizumab, leading to delayed detection of the event.^[13]^ While a few diverticula were observed in the ascending colon in the present case, there were no findings of diverticulitis. Thus, the intestinal mucosal injury was not associated with diverticular disease in this case, indicating the rarity of this condition. A search of the PubMed database revealed 4 case reports of tocilizumab-induced intestinal mucosal injury in patients who underwent endoscopy (Table 2).^[6–9]^ These cases were unrelated to diverticulosis. Regarding the primary disease, 3 cases were of RA, 1 case was of COVID-19, and only our case was of AOSD. Four of the 5 patients presented with hematochezia, and 2 presented with abdominal pain. The time from tocilizumab initiation to symptom onset varied (14 days to 9 months). Four patients had lesions in the ileum and 3 had lesions in the colon. Most of the patients had ulcerative lesions. Punched-out ulcers were seen in 2 cases, including the present case. Only one case had a perforated lesion. Tocilizumab discontinuation was performed in 3 cases, and surgery was performed in 2 cases. All patients were alive after treatment. To our knowledge, this is the first case of tocilizumab-induced small intestinal injury that was treated endoscopically for gastrointestinal bleeding.

**Table 2:** Case reports of tocilizumab-induced ulcer.

Case	Author/Yr	Age/Sex	Primary disease	Chief complaint	Duration of TCZ therapy	Location	Endoscopic findings	Treatment
1	^[12]^/2011	57/F	Rheumatoid arthritis	Hematochezia	3 mo/2 wk	Ileum, colon	Aphthoid ulcer/punched-out ulcer	Discontinuation of TCZ, fasting
2	^[13]^/2020	43/M	COVID-19	Diarrhoea, hematochezia	16 d	Ileum	Erythematous mucosa, large ulcer	Operation
3	^[14]^/2022	74/F	Rheumatoid arthritis	Hematochezia, abdominal pain	6 mo	Cecum to transverse colon	Multiple irregular ulcers	Discontinuation of TCZ
4	^[3]^/2022	29/M	Rheumatoid arthritis	Diarrhoea, abdominal pain	14 d	Ileum, cecum, ascending colon	NA (perforation)	Operation
5	Our case	64/F	Adult-onset Still disease	Hematochezia	9 mo	Ileum	Punched-out ulcer	Discontinuation of TCZ, fasting

COVID-19 = coronavirus disease 2019, NA = not available, TCZ = tocilizumab.

Diagnostic criteria for tocilizumab-induced mucosal injury have not been established, and the characteristics of the lesions are unknown. In the present case, the tocilizumab-induced lesion was a deep punched-out ulcer resembling intestinal Behçet’s disease. In our case, a colonoscopy was performed for hematochezia, and we suspected intestinal Behçet’s disease based on the location and shape of the lesion. However, the ulcer in the ileum reduced in size over time, indicating that it is a drug-induced ulcer and not intestinal Behçet’s disease. Furthermore, discontinuation of tocilizumab resulted in rapid ulcer scarring, which led to the diagnosis of a tocilizumab-induced small intestinal ulcer. Iwasa et al also reported the case of a RA patient with punched-out ulcers in the lower gastrointestinal tract during tocilizumab therapy.^[6]^ In this case, the multiple ulcers that were cured by discontinuation of tocilizumab recurred after the re-administration of tocilizumab, strongly supporting a diagnosis of tocilizumab-induced mucosal injury. Hence, when a patient on tocilizumab therapy presents with punched-out ulcers in the small intestine, tocilizumab should be withdrawn to account for drug-induced mucosal injury, and additional immunosuppressive therapy should be avoided for successful treatment.

Although the precise mechanism of tocilizumab-induced mucosal injury remains unclear, it is hypothesized that one of the effects of IL-6 blockade is the decrease in T helper 17 (Th17) and T helper 22 (Th22) cells; IL-6 is essential for the differentiation of Th17 and Th22 cells. Th22 cells are a recently identified subset of cluster of differentiation 4 (CD4)-positive T cells that differentiate from naive CD4-positive T cells in the presence of IL-6. Th22 and Th17 cells are known to produce IL-22, which acts in mucosal defense, tissue repair, and wound healing.^[14]^ Therefore, tocilizumab, a monoclonal anti-IL-6 receptor antibody that interferes with IL-6 signaling, may prevent naive CD4-positive cells from differentiating into Th17 and Th22 cells, resulting in mucosal injury.

In recent years, tocilizumab has also been used to treat COVID-19. It is known that IL-6 is elevated in patients with severe COVID-19 owing to an excessive immune response, which correlates with disease severity;^[15]^ thus, the usefulness of tocilizumab as a therapeutic agent against COVID-19 has been investigated. In the RECOVERY trial conducted from April 2020 to January 2021, the 28-day mortality rate of COVID-19 patients with hypoxemia was significantly lower in the tocilizumab group than in the placebo group (31% vs 35%, risk ratio 0.85, 95% confidence interval 0.76–0.94, *P* = .0028).^[16]^ As the COVID-19 pandemic has not been sufficiently contained and the use of tocilizumab is expected to increase, it should be widely recognized that tocilizumab can induce intestinal mucosal injury. Tocilizumab can cause deep ulcerative lesions in the small intestine, resembling intestinal Behçet’s disease. It is important to continuously monitor abdominal symptoms during tocilizumab therapy and aggressively perform endoscopy when hematochezia or abdominal pain is observed.

However, there was no direct evidence that tocilizumab caused the small intestinal ulcers in our case. In other words, if the lesion were to reoccur after re-administering tocilizumab, it would strongly suggest a diagnosis of tocilizumab-induced mucosal injury. However, this could not be attempted for ethical reasons. Therefore, it is important to carefully rule out other causes of small intestinal ulcers.

In conclusion, we report a case of AOSD with tocilizumab-induced intestinal mucosal injury. Tocilizumab can cause punched-out ulcers in the small intestine, mimicking intestinal Behçet’s disease. This suggests that small intestinal ulcers should be considered as a differential diagnosis when hematochezia and abdominal pain are observed during tocilizumab therapy. In addition, in cases of properly diagnosed tocilizumab-induced ulcers, unnecessary immunosuppressive therapy should be avoided, and tocilizumab should be discontinued.

## Author contributions

**Conceptualization:** Sotaro Ozaka, Koshiro Tsutsumi, Kazunari Murakami.

**Data curation:** Masahide Fukuda.

**Supervision:** Haruhiko Takahashi, Masao Iwao, Yuka Hirashita, Kensuke Fukuda, Kazuhisa Okamoto, Mie Arakawa, Ryo Ogawa, Mizuki Endo, Kazuhiro Mizukami, Naganori Kamiyama, Masaaki Kodama, Kazunari Murakami.

**Writing – original draft:** Sotaro Ozaka.

**Writing – review & editing:** Takashi Kobayashi.
